# Safety Management Practices in Small and Medium Enterprises in India

**DOI:** 10.1016/j.shaw.2014.10.006

**Published:** 2014-11-04

**Authors:** Seema Unnikrishnan, Rauf Iqbal, Anju Singh, Indrayani M. Nimkar

**Affiliations:** 1 Industrial Safety and Environmental Management Group, National Institute of Industrial Engineering (NITIE), Vihar Lake, Mumbai, India; 2 Center for Environmental Studies, National Institute of Industrial Engineering, NITIE, Vihar Lake, Mumbai, India

**Keywords:** best practices, safety, small and medium sized enterprises

## Abstract

**Background:**

Small and medium enterprises (SMEs) are often the main pillar of an economy. Minor accidents, ergonomics problems, old and outdated machinery, and lack of awareness have created a need for implementation of safety practices in SMEs. Implementation of healthy working conditions creates positive impacts on economic and social development.

**Methods:**

In this study, a questionnaire was developed and administered to 30 randomly chosen SMEs in and around Mumbai, Maharashtra, and other states in India to evaluate safety practices implemented in their facilities. The study also looked into the barriers and drivers for technology innovation and suggestions were also received from the respondent SMEs for best practices on safety issues.

**Results:**

In some SMEs, risks associated with safety issues were increased whereas risks were decreased in others. Safety management practices are inadequate in most SMEs. Market competitiveness, better efficiency, less risk, and stringent laws were found to be most significant drivers; and financial constraints, lack of awareness, resistance to change, and lack of training for employees were found to be main barriers.

**Conclusion:**

Competition between SMEs was found to be major reason for implementation of safety practices in the SMEs. The major contribution of the study has been awareness building on safety issues in the SMEs that participated in the project.

## 1 Introduction

Small and medium enterprises (SMEs) are important to almost all economies in the world, especially in developing countries. In developing countries SMEs constitute the middle size range, which explains their strategic importance and their output share can be greater or less than its employment share. The size and importance of the SME sector varies from country to country; the last few decades have seen an increasing recognition of the role it plays in industrial countries due to which number of SMEs is increasing [1,2]. SMEs alone contribute to 7% of India's gross domestic product (GDP). They constitute 90% of the industrial units in the country and also contribute to about 35% of India's exports [3]. The SME sector of India is considered as the backbone of economy contributing to the industrial output (45%), exports (40%), giving employment to about 60 million people, creating 1.3 million jobs every year and producing more than 8,000 products for the Indian and international markets. Many factors are responsible for the growth of Indian SMEs including funding to SMEs, the new technology and various trade directories and trade portals [4].

The micro, small, and medium enterprises (MSME) sector accounts for 45% of the manufacturing output and 40% of the total exports of India. In 2013 the total number of enterprises in MSME sector was estimated to be 36.2 million, of which 1.6 million were in the registered sector and 34.6 million enterprises in the unregistered sector, with a total employment of 80.5 million. Uttar Pradesh is the leading state of India in terms of enterprises (4.4 million) and employment (9.2 million). In the MSME sector of India, rural area and urban area have 20.0 million and 16.2 million working enterprises, respectively. 31.79% of the enterprises are engaged in manufacturing whereas 68.21% of the enterprises are engaged in the services [5].

In this rapidly globalizing world, safety performance is a key issue for the industries to become a world-class competitor. Occupational accidents may lead to permanent disabilities or deaths and/or economic losses or both [6]. Occupational accidents can be reduced through effective preventative measures by hazard assessment, good housekeeping, training, and better personal protective equipment (PPE) [7]. In order to develop a good safety culture, the attitude of the workers needs to be reoriented by adopting best practices, good housekeeping, and changes in work culture and work practices. Occupational accidents are common in India, as in many other developing countries. Prediction of various types of accidents helps managers to formulate organizational policies for improving safety performance [8].

In the organizational context, technology innovation may be linked to performance and growth through improvements in efficiency, productivity, better safety through proper human factor design, environmental quality, etc. Technology innovations in SMES are possible in the design of products, processes, supply chains, etc. [9]. Unlike the organized sectors, SMEs are not equipped with sophisticated technology, structured environment, or safety and health practices. Often in an SME, workers need to work in adverse working conditions [10]. This leads to accidents, injury, and product loss.

Every employer has a responsibility towards each employee to ensure, as far as is reasonably practicable, that the employee is, while at work, safe from injury and risks to health. An employee's perception will reflect how they believe that safety is to be valued in the organization [11]. Top management is often responsible for the implementation of safety-enhancing systems and the development of a safety-oriented culture [12]. Komaki et al [13] studied the impact of worker behavior on safety and concluded that training and reinforcement of safety practices help in preventing accidents on the workfloor. They also suggested that in-house safety programs are ineffective without systematic assessment. Safety consciousness refers to an individual's own awareness of safety issues [14].

This awareness works on both a cognitive and a behavioral level. Behaviorally, safety consciousness enacts the behaviors that foster operational safety. Inspirational motivation (communicating a safety-oriented vision) communicates the importance of safety and motivates employees to care about safety. It raises awareness of safety issues and also motivates them to enact behaviors oriented on safety. Intellectual stimulation (challenging employees to think of new ways to improve safety) causes employees to think about what behaviors could improve safety and broadens their knowledge base regarding safety-oriented behaviors [12].

The pioneering work of improving workplace safety utilizing behavioral approaches to safety was done by Komaki et al [15]. Their study reported that behavioral safety programs encouraged employees to act safely. Similar findings were echoed by studies of Cooper et al [16], Krause et al [17], and Cox et al [18]. Safety policy refers to the extent to which a senior manager creates a clear mission, responsibility, and goal in order to set standards of behavior for employees; and sets up a safety system to correct workers' safety behaviors. Safety concern refers to the extent to which a senior manager stresses the importance of safety equipment, emphasizes their interests in acting on safety policies, is concerned about safety improvement, and coordinates with other departments to solve safety issues [19].

Safety leadership motivates team members to work harder, to work efficiently, and to take ownership of responsibility for safety performance [20]. The Health and Safety Executive has stated that without effective leadership one cannot have good safety performance. The Federal Safety Commissioner [21] also emphasized the importance of safety leadership of senior managers in achieving a safety culture. The increasing attention being paid to safety leadership in various industries is the evidence of the assumption that safety leadership will result in increased organizational safety effectiveness [19].

Developing and sustaining safety leadership is important to reduce accidents and to promote safety among managers and general employees. Leadership has been fully implicated in safety, with the majority of previous studies examining the full-range model of transformational and transactional leadership behaviors in managers and supervisors [14,22,23]. Wu et al [24] defined safety leadership as “the process of interaction between leaders and followers, through which leaders can exert their influence on followers to achieve organizational safety goals under the circumstances of organizational and individual factors”.

Safety culture is a subcomponent of organizational culture, which considers affecting members' attitudes and behavior in relation to an organization's ongoing health and safety performance [25]. The term *safety culture* first made its appearance in the 1987 OECD Nuclear Agency report [26] (on the 1986 Chernobyl disaster). Safety culture is frequently identified, for example by disaster inquiries, as being fundamental to an organization's ability to manage safety related aspects of its operations successfully or otherwise. Safety culture comprises attitudes, behaviors, norms, and values, personal responsibilities as well as such HR features as training and development [27].

The safety culture concept grows as it absorbs streams of learning from diverse research and organizational sources. The safety culture concept, because of its possibilities and ambiguities, is proving to be a stimulus for many to gain a deeper understanding of the modern technological organization as a complex system with many interactive and adaptive features. It also reveals the progress that is being made and the challenges to be faced, as researchers and practitioners strive to make the concept meaningful for organizations that wish to use behavioral change as a means of improving safety performance [28].

Pousette et al [29] refer to safety climate dimensions, such as management safety priority, safety management, and personal involvement, all of them respecting the considered dimensions of work environment and personal motivation. The holistic as well as the shared aspect of culture and climate are stressed in most definitions with terms such as *molar* [30,31], *shared* [32–34], *summary* [35], *group* [36], *set* [37], *assembly* [38], *employees' perceptions*, or *organization's beliefs and attitudes* [39–41]. Safety climate could also be an important predictor of safety behavior. Given the organizational nature of the safety climate, some authors argue that safety climate could be related with the company, or organization size. As suggested by Zohar [42], workers' safety (perceived) climate plays an important role in increasing the percentage of safe actions, such as the use of hearing protection devices.

Carrillo and Simon [43] proposed the Safety Culture Leadership Inventory, which comprises six critical leadership practices: to make the case for change, to create a shared vision, to build trust and open communication, to develop capabilities, to monitor progress, and to recognize accomplishments.

The main objective of this paper is to study the safety management practices in SMEs of India. A secondary aim is to evaluate the safety practices and benchmark with the best practices in that particular sector. Also, this paper helps to understand the drivers and barriers for change and the status of environment, safety and health in the SMEs in different states of India.

## 2 Materials and methods

The study was carried out in 30 SMEs located mainly in Mumbai, Maharashtra, and a few other states in India. The SMEs were randomly chosen to evaluate safety practices. Also, the study looked into the barriers and drivers for technological innovation and recommended best practices on safety issues.

Groups of students selected SMEs based on their willingness to participate in their survey. The content of the questionnaire was decided with input from process safety experts. Observations were used to fill up data in some units.

Primary data collection was done in 30 units in 2013–14 and secondary data were collected from reports from organizations such as World Health Organization, Ministry of MSMEs, electronic data bases such as ScienceDirect, Wiley, and Open Access Journals.

For this study, a questionnaire was developed for capturing the data having both open- and close-ended questions. Stepwise methodology is given below:

• Visit of selected industries for primary data collection
• Study and evaluate the safety impacts of existing data collection on:
  ○ Technology details
  ○ Accident scenarios [unsafe conditions, isolated storage, tools, and chemicals used]
  ○ Various safety hazards
• Secondary data collection of the best technologies in the sector and comparative analysis of data
• Advising industries on the best possible technology and practices from the safety point of view.

The questionnaire was used for primary data collection and divided into four sections as follows:

Section A: About the firm

1. General information
2. Financial aspects
3. Manpower

Section B: Details of technology used

1. Technology details
2. Process flow
3. Historic data for safety issues in technology
4. Main product and by-products
5. Adverse impacts of technology on human health, water quality, noise level, energy efficiency (rate on scale 7–highest, 1–least)
6. Factors required for encouraging safety practices

Section C: Evaluation of safe and clean technology

1. Adverse safety impacts
2. Accident scenarios
3. Near misses during last 3 years
4. Credible scenarios during last 3 years
5. Catastrophic scenarios during last 3 years

Section D: Unsafe conditions, isolated storage, tools, and chemicals used

1. Risk increased and decreased in last 3 years, with reasons
2. Drivers of safe technologies
3. Barriers of safe technologies
4. Recommendations for safe technologies

A Likert scale was used for the questionnaire, where a ranking of 1 means real near miss reporting is less and 5 means people are actually reporting near misses, giving it importance. The main study material used is the questionnaire with the above section used for collecting primary data from SMEs.

### 2.1 Background information of the units

A total of 30 units from different sectors across India were visited to obtain information about their turnover, numbers of employees, mode of operation, and safety management practices through an interviewer.

**Fig. 1:**
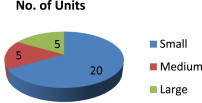
Distribution of units (Rupees) according to scale.

**Fig. 2:**
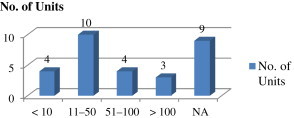
Turnover range of the units (million Rupees). NA = no data given.

Fig. 1 describes the scale-wise distribution of units visited. Out of 30 units visited, 20 were small scale, five were medium scale, and five units were large scale. Fig. 2 describes the turnover of the units visited. Out of the visited 30 units, 10 units had a turnover of 11–50 million, four units each had turnover < 10 million and 51–100 million, and three units had a turnover > 100 million; nine units did not mention their turnover.

**Fig. 3:**
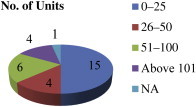
Number of employees.

**Fig. 4:**
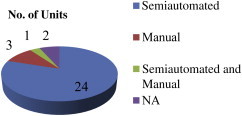
Mode of operation.

Fig. 3 gives the employment details of the visited units. Among the visited industries very few (4) had > 100 employees and 15 had < 25 employees. Fig. 4 provides the information about mode of operation in the units. Out of 30 units, 24 units were semiautomated, three had manual operations, and one unit operated using both manual and semiautomated systems. Two units did not give details about their operations.

**Fig. 5:**
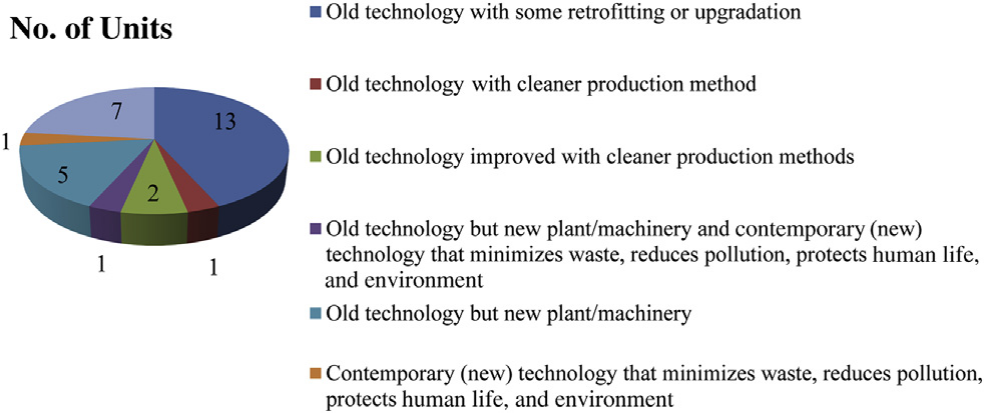
Technologies used in units.

Fig. 5 presents the distribution of different technologies used in visited units. It was observed that old technology with some retrofitting or upgrading was used in 13 units followed by old technology, but new plant/machinery was used in five units. Old technology improved with cleaner production methods was used in two units, whereas old technology but new plant/machinery along with contemporary (new) technology that minimizes waste, reduces pollution, protects human life and environment, and old technology with cleaner production methods were used in one unit each. Seven units did not mention their technology. As small-scale industries may have financial problems, most units use old technologies with some upgrading or modification.

## 3 Results

### 3.1 Evaluation of safe and clean technology

The safety impacts were evaluated using a 7-point Likert scale. The findings about the adverse safety impacts are summarized in Table 1, which shows that safety impact was ranked as high in five units. It was also seen that near miss reporting was less, as only five units ranked near miss reporting as high.

In 12 units we can see that no data have been maintained in the credible scenario, which may be based upon either actual experiences or historical experiences of an industry; 20 units have very low rating for catastrophic scenarios such as major accident, explosions, major fire, and economic collapse. Using old technologies, growth in the business, aged workforce and less concern for environment, and health and safety standards have increased the risk factors on the workers and productivity.

As per the survey (Table 2) out of 30 units, 16 mentioned an increase in operational risks in past 3 years and six mentioned a decrease in operational risks in the past 3 years due to use of safety practices in the operations; one unit mentioned both an increase and decrease in operational risks. Eight units did not have any kind of data regarding increases or decreases in operational safety. It can be seen from the survey that the risk has increased in many SMEs, the reasons being lack of training workers, increase in the size of the workforce, and negligence of workers towards their safety. As employees grow older, and if the work requires a very high level of precision, the risk becomes higher. Injuries such as thumb and leg injury, and some minor cuts are common. In a few SMEs the risk decreased due to the use of new technologies. Ergonomic risks have reduced due to automation of some of the machines, formation of safety committees, machine guarding, etc. It can be seen that SMEs with low financial budgets have not taken major steps towards safety practices and some SMEs with healthy financial budgets have initiated the safety practices in their units.

### 3.2 Drivers and barriers for safe and clean technology

Out of 30 SMEs only 15 responded regarding drivers and barriers for safe and clean technology; the remaining 15 stated that there are no significant drivers and barriers to report. The drivers and barriers for safe and clean technology faced by the SMEs were analyzed based on responses obtained through the survey and accordingly the most significant drivers (as per Table 3) for safe and clean technology and use of the latest and upgraded technology were market competitiveness, better efficiency, less risk, and stringent laws. Leadership commitment and senior management motivation are the other important motivators for such practices. The main barriers to introduction of new safe and clean technology were financial constraints as management is not ready to invest a huge amount as these are small enterprise and may not be able to earn any profit due to such investment. Lack of awareness, resistance to change, and lack of training for employees in the field of safety are other barriers.

From the study it was revealed that competition between the SMEs was found to be a major reason for implementation of safety practices in the SMEs. Thus, top management commitment was found to be more useful in order to manage safety in the workplace.

In this survey, the selected SMEs were evaluated for the safety management practices implemented in their facility. As per the survey, we can say that these SMEs are at the initial stage of implementing safety practices and have not reached a sufficient level; some of them have started initiating safety practices in their unit.

### 3.3 Best safety practices emerging from the survey

We collected primary data from SMEs to improve safety practices as one of the questions asked in the survey was based on their experiences, and secondary data were collected from open access websites and journals based on best practices in safety.

Table 4 shows recommendations for best safety practices for SMEs, which have been segregated using different facilities/operations in the industries such as machine operation, welding and cutting operation, hand tool operation, grinding dust and hazardous fumes, electrical work, fire safety, storage of materials, manual handling, housekeeping, and PPE along with the hazards associated with the operations and recommendations on the respective hazard.

As per the responses obtained from the survey the recommendations suggested for improving the operations include the use of PPE, safety interlocks on high temperature and pressurized machines, use of guards, interlock switches, and dead man's handles, regular services and maintenance of all the equipment and machines, use of a respirator, regular service of electrical equipment, proper storage of raw materials and products, use of serviced and certificated fire-fighting equipment, clear signposting, unobstructed and unlocked fire exits and escape routes, frequent and random fire drills, and storage of flammable, combustible, toxic, and other hazardous materials in approved containers in designated areas.

In the present study, it was observed that the safety management practices are inadequate in most of the SMEs. Therefore, there is need to improve management practices to enhance safety standards, which will lead to better productivity.

## 4 Discussion

### 4.1 General discussion

In this survey, we gathered information on different safety practices followed in the surveyed units such as well-defined safety goals, documentation of safety policies, green purchasing policy, safety standards for suppliers, safety audits at regular intervals, internal safety standards. It was observed that few units have safety standards for their suppliers; they perform safety audits at regular intervals and have internal safety standards. Very few units have well-defined safety goals, documented safety policy, or green purchasing policy. Out of 30 units, one unit had implemented all the aspects of environmental friendly practices, whereas many units were found not following any kind of such practice. Some of the reasons for the negative responses towards following safety practices were lack of awareness along with financial constraints, lack of interest from management side, concern only towards productivity, purchase as per client's specifications, finding implementation of policies expensive and infeasible, and fewer operating margins to conduct external audits.

To collect the primary data, random and convenient sampling was done using a questionnaire survey from the SMEs who were willing to participate in the survey. The main limitation to our study was that we covered only 30 SMEs. We captured clear barriers and drivers using an open-ended questionnaire. To evaluate the safety impacts we used a 7-point Likert scale methodology. For research based on survey questionnaires a Likert scale used as a psychometric scale, wherein the respondents indicate their level of agreement or disagreement on a symmetric scale for a series of statements [44]. Some of the advantages of a Likert scale are that it has good reliability, can be easily generated and modified, and the outcome can be directly used for statistical implications. The disadvantages of a Likert scale are that it fails to estimate intervals of ordinal data, and the respondents are forced to make a choice from the given options that may not match their exact responses [45].

Behavior-based safety (BBS) focuses on the identification and modification of critical safety behaviors, and emphasizes how such behaviors are linked to workplace injuries and losses. There is a specific technology derived largely from operant psychology that can be drawn upon to develop, implement, and evaluate BBS programs in various work settings [46]. Communication has consistently been identified as a key element of safety program effectiveness [47,48], safety behavior change [49], safety training effectiveness [50], and safety culture/climate [51]. Research indicates that BBS has reduced accident rates by 40–75% within 6–12 months of its implementation [52]. BBS training is found to facilitate a growing number of safe behaviors, help reduce the number of unsafe behaviors, and assist in decreasing the number of unsafe conditions in the organization [53]. A well-planned and implemented behavioral safety system such as BBS can instill workforce stewardship of safety systems and lead to fewer accidents, incidents, near-misses, and property damage; acceptance of the safety systems; and increased reporting of defects, near-misses, and accidents [54].

In this study we have covered different sectors and this has led to lot of awareness building in SMEs. In many SMEs, small changes were implemented immediately, such as better housekeeping and use of PPE. The best practices were recommended based on inputs given by the SMEs as one of the questions asked them was to give recommendations to improve safety practices based on their experiences and secondary data were collected from open access websites and journals based on best practices in safety.

We have consolidated the safety practices primary and secondary data and forwarded the recommendations to respective SMEs. In future we intend to go back to the studied SMEs for follow up.

### 4.2 Practical implications and best practices

Safety issues in the process technology as per survey are as follows:

• Encouragement by employers to use PPE even during small operations, regular counseling and audits at regular intervals by the officials, and adopting and maintaining the standard operating procedures for every operation carried out in the unit.
• Adequate inspection and testing of electrical installations and equipment.
• Proper housekeeping, such as removal of metal scrap to avoid any accidents. Scrap should be sold only to government-certified scrap disposal companies. Most of the units have also recommended implementation of *5S*, i.e. sorting (*Seiri*), streamlining (*Seiton*), systematic cleaning (*Seiso*), standardize (*Seiketsu*), and sustain (*Shitsuke*).
• In working sites, the safety and environmental policy must be written down and maintained regularly.
• Some individual units cannot afford Safety Officers; the complex consisting of many MSMEs can together fund a safety officer, to be made mandatory as per law.
• Ergonomics suggestions given by the executives must be taken into consideration for safe working in the unit. The important ones are taking care to see that the working posture of the employees is proper and comfortable. While working with a hammer, some employees were sitting on the floor surrounded by finished products and a machine; a proper workplace should be provided. The lighting condition of the working area for vacuum varnishing was not adequate; the workplace should be supplied with more lighting sources.
• Units that deal in chemical operations have recommendations about bulk storage as a possible safety issue.
• Adoption of Business Continuity Management standards.
• Organization of outside help and mutual aid such as agreements with the local police, fire department, and hospitals.

## 5 Conclusions

The major contribution of this study has been an insight into SMEs' perspectives on safety and awareness building on safety issues in participants. In many units, some simple recommendations that could be easily implemented without high capital cost, such as improved housekeeping, better layout, and using PPE were put into practice.

It was noted during our visits that in many units there has been an increase in the risk due to overproduction and crammed areas as the majority of SMEs are using old technologies. Minor injuries are quite common in various units and the employees do not consider it to be a matter of serious concern. Studies such as this, undertaken only for SMEs, are extremely important to improve safety awareness and practices.

## Conflicts of interest

There is no conflict of interest. The small and medium scale enterprises who have participated in the study are not competing with each other. The authors are academicians who will not be affected by the outcomes of the study and all of us are unanimous in our publishing decision.

## Figures and Tables

**Table 1:** Summary of adverse safety impacts (from survey)

No. of SMEs reporting adverse safety impacts				Rating
Safety impact	Near misses	Credible scenario	Catastrophic scenario	
0	0	0	0	Very high
5	5	0	0	High
13	3	3	0	Medium
2	5	7	1	Low
3	7	8	20	Very low
7	10	12	9	NA

**Table 2:** Historical data of the past 3 years and increases or decreases in risks during the past 3 years in the 30 small and medium enterprises studied

No.	Company	Safety issues in the process technology	Risks increased or decreased in last 3 y
1	SME-1 Engineering	• Minor accidents like small cuts and bruises • Ergonomics problems	Risk increased due to: • High risks as employees are old age. • Competition • Sizes of the unguarded machineries increase the risks during daily operations
2	SME-2 Engineering	• Old and outdated machinery • Safety practices are not in place for workers	Risk increased due to: • Overstress and increased loads • The limitation of space leading to more accidents
3	SME-3 Trading company	• Improper handling of galvanized iron sheet roll	Risk increased due to: • Increase in the business • Material storage space • Material on manufacturing floor
4	SME-4 Engineering company	• Very few minor injuries due to negligence of workers	Risk decreased due to: • Automation of some of the machines
5	SME-5 Engineering unit of metal works	• Minor injuries to workers	Risk decreased due to: • Installation of semiautomated bending machine
6	SME-6 Printing	No record maintained	Insufficient records
7	SME-7 Construction	No data available	Insufficient records
8	SME-8 Motor company	No data available	Insufficient records
9	SME-9 Motor manufacturing	• Improper working condition • Improper housekeeping • Inadequate ventilation • Inadequate waste collection and disposal	Risk increased due to: • Insufficient processing areas • Inadequate firefighting equipment • Haphazard electrical wiring • No use of personal protective equipment (PPE)
10	SME-10 Engineering	• Electrical safety issues	Risk increased possibly due to: • No use of PPE
11	SME-11 Processing unit	• Maharashtra State Electricity Board unit transformer fire, scrapyard fire • Leg injury to workers	Risks decreased due to: • Formation of safety committee • Installation of extinguisher, fire hydrant • Machine guarding and automation
12	SME-12 Plastics	Safety issues for workers	Risk increased possibly due to: • Improper isolation of heating chamber • Workers operating in low illumination • Limited use of PPE • Limited walking space and absence of emergency exit
13	SME-13 Waste management facility	No record maintained	No record maintained
14	SME-14 Bread factory	Safety issues for workers • Absence of records • Poor illumination • Poor house keeping • Poor machine design	Risk increased possibly due to: • Absence of fire extinguishers • Absence of emergency exits • Poor safety culture
15	SME-15 Graphics unit	• Handling of organic liquids • Moving parts in Lathe machine • Fire in the factory premises	Risk decreased due to: • Use of PPE
16	SME-16 Leather gallery unit	Nil	No record maintained
17	SME-17 Leather unit	• Leather dust accumulation • No machine guarding • Inadequate number of drills	No accident in past 3 y of operationRisk may increase in future due to: • Improper maintenance of machines
18	SME-18 Packaged drinking water	• Absence of emergency coordinators • Absence of emergency plan (fire and natural disasters) • No emergency lighting • No mutual aid agreement with local bodies and police	Risk increased possibly due to: • No training • No standard operating procedure (SOP) for shut down operations • Narrow shop floor area • No emergency exit • No fire alarm and sprinkler system
19	SME-19 Bakery unit	• No emergency lighting	Risk increased possibly due to: • No SOP for shut down operations
20	SME-20 Plastic unit	• Absence of emergency coordinators • Absence of emergency plan • Loose wiring • Poor maintenance of electrical boxes	Risk increased possibly due to: • No SOP for shut down operations • No training to workers • No use of PPE • No emergency exit • No fire alarm and sprinkler system • Narrow shop floor area
21	SME-21 Construction unit	No record maintained	No accident in past 3 y of operation.Risk decreased possibly due to: • Safety consciousness • Use of PPE • Use of newly developed laminates reduced the risk of getting exposed to toxic fumes.
22	SME-22 Rubber unit	No record maintained	No record maintained
23	SME-23 Logistics unit	• Collision with forklifts • Falling from height • Falling objects • Slips, trips, fall • Chemical spills • Inhaling of battery fumes	No record maintained
24	SME-24 Metal alloy unit	No record maintained	No accident in past three years of operation.Risk might increase in future possibly due to: • Manual operations • Importance of safety is not recognized in the factory.
25	SME-25 Food unit	No record maintained	Risk decreased due to: • Excluding flies by using double-door entry • Use of acrylic tube light sheets
26	SME – 26 engineering unit	• Finger injury	Risks increased due to: • Manual handling of components at press shops
27	SME-27 Hydraulic unit	No accident in past 3 y of operation.	No major accident in past 3 y of operation. Risks increased due to: • Manual operations • Importance of safety is not recognized in the factory.
28	SME-28 Tyre retreading unit	• Hot burns • Foot injury resulting	Risk increased due to: • Human error • Old technology with some retrofitting/upgrading Risk decreased due to: • Better storage of raw material happen in reduction of fire/spill risk
29	SME-29 Electrical unit	• Old construction of plant	Risk increased due to: • Use of old machines
30	SME-31 Roadways unit	No record maintained	No record maintained

**Table 3:** Drivers and barriers for safe and clean technology

No.	Company	Drivers	Barriers
1	SME-1 Engineering Unit	• Competitors using efficient, safe and clean technologies. • Frequent accidents causing employer to pay medical charges	• Low encouragement by employer • Ignorance of the management and workers • Lack of clean and green technology
2	SME-2 Engineering	• Competitors using efficient, safe and clean technologies	• Lack of funds and awareness
3	SME-4 Engineering company	NA	• Lack of funds and awareness
4	SME-5 Engineering unit of metal works	• Customer demand	• Lack of funds • High cost of technology
5	SME-6 Printing	• Customer demand	• High cost
6	SME-7 Constructions	• Competitors using efficient, safe and clean technologies. Apart from that, in order to finish the work with good quality the company has to use good technologies that obviously increase the degree of technology in the aspects of safety and cleanliness.	Money is the main barrier of the safe and clean technologies. Even though the top management insists on using good technologies, the ignorance of the workers in that field nullifies the efforts of the management.
7	SME-8 Motor Company	Nil	The main barriers for introduction of new safe and clean technology are: • Resistance to change • Ignorance • Financial constraints Lack of training for employees in field of safety
8	SME-10 Engineering	• The most important thing with which the occupier is concerned is with the safety of the workers. • For that the machines have been provided with the good illumination and leg operating switches. This helps in on the spot start and stop feature so as to avoid any design glitches as well as safety issues. • The main driver here is the work safety because in the past too there have been incidences of minor injuries due to cutting with open hands.	• The enforcement agencies are not concerned of such small units adopting clean and safe technologies. • Workers are not inclined to follow safe practices as they are accustomed to work in that particular manner since a long time now. To bring such a drastic change will need a paradigm shift, which in such a small unit is virtually impossible
9	SME-11 Processing unit	Installation of safety devices, effluent treatment plant, automatic trip indicators, technological up-gradation, regular safety audits	There are no major barriers
10	SME-12 Plastics	• Use of safe technology is directly proportional to worker's productivity	• Lack of administrative measures and controls • Insufficient financial and technical resources to invest in safety improvements • Easy availability of work force who does not demand a safe and clean working environment • Industry does not need such measures as the existing ones are serving their purpose
11	SME-13 Waste management facility	Leadership commitment and senior management motivation	Nil
12	SME-16 Leather gallery unit	Nil	Lack of awareness regarding safety and ergonomics issues
13	SME-17 Leather Unit	Nil	Since no accidents have taken place in the past the complacent attitude of the owner is the main barrier
14	SME-29 Electrical unit	NIL	Economic constraint
15	SME-31 Roadways Unit	• Reputed clients. • Caring for its own workers, so zero accident record is maintained and loss hours of work is prevented	• Management is not interested in investing in safer technologies as no hazardous chemicals are stored in the warehouse • Complacency as there has not been any accident in the warehouse so far

**Table 4:** Recommendations for safety best practices for small and medium enterprises

No.	Equipment/facility	Hazards	Recommendations
1	Machine operation	In-running nips, moving parts, risk of cut, crush	• There must be safety interlocks on high temperature and pressurized machines • Use of guards, interlock switches, and dead man's handles to ensure the machines cannot be operated when moving parts are exposed • Machines must undergo regular servicing and maintenance
2	Welding and cutting operation	• Gas welding and cutting tools are often powered by oxygen or acetylene gas cylinders. These tanks require special safety precautions to prevent explosions and serious injuries. • Metal fumes, radiation, hot metals and noise	• Use of PPE • General ventilation and exhaust system • Ensure that acetylene/oxygen systems are equipped with flame or flashback arrestors. Store acetylene bottles upright and secured • Set acetylene pressure at or below 15 psi. Always use the minimum acceptable flow rate. Never use a match to light a torch. Use an approved lighter.
3	Hand tool operation	Excessive use of hand tools is associated with chronic disorders of the hand, wrist and forearm, such as carpal tunnel syndrome and wrist tendonitis	• Hand tool should match the task that the user is doing • Hand tool design should: ○ Reduce the force of application ○ Fit the users hand ○ Can be used in a comfortable position • Hand tools should be well maintained
4	Grinding dust and hazardous fumes	• Very dangerous to health, especially beryllium or parts used in nuclear systems • Inhalation of the dust and fumes goes into the lungs and mixed with blood • Effect is temporary sickness to death	• Use of respirator to avoid inhaling the dust. Use of coolant during grinding • These materials require careful control of grinding dust
5	Electrical work	Short circuits caused by wear and tear and poor servicing	• Lock out and tag out • Regular maintenance of equipment and machines
6	Fire safety	Fire hazard	• Electrical equipment must be regularly serviced • Combustible materials must be stored safely • There must be adequate and appropriate firefighting equipment • Firefighting equipment must be serviced and certificated • Fire alarm points must be clearly signed and accessible • Fire exits and escape routes must be clearly signposted, unobstructed and unlocked • There must be a fire assembly point a safe distance from the factory, with frequent, random fire drills carried out • Smoking must be banned in working areas of the factory
7	Storage of materials	Slip, trip, fall, fire hazard	• The location of the stockpiles should not interfere with work • Stored materials should allow at least one meter of clear space under sprinkler heads • Stored materials should not obstruct movement • Storage areas should be clearly marked • Flammable, combustible, toxic and other hazardous materials should be stored in approved containers in designated areas
8	Manual handling	Acute and chronic injuries, slip disc, musculoskeletal disorders (MSDs) and other types of injury	• Not exceeding load lifting limit • Designing proper work rest schedule • No employee should be required to routinely work above their shoulder height, below their knees or at full reach distance
9	Housekeeping	Poor housekeeping can result in an increased risk of injury due to slip, trips and falls, together with injuries resulting from hitting stationary objects, are reduced	• Areas to be kept clean and free for movement • Items should be stored correctly with no parts protruding onto walkways • Electrical cords should not be on the floor • Tools should have designated areas for storage and bins for waste should be readily available and be easy to empty
10	Personal protective equipment (PPE)	Inadequate unavailable	Appropriate PPE must be provided and worn. Wherever possible, the need for PPE should be removed by automating or using engineered safety features on machinery (such as interlock switches)

## References

[1] Berry A. (2007). The importance of SMEs in the economy.

[2] Keskìn H., Sentürk C., Sungur O., Kìrìs H.M. (2010). The importance of SMEs in developing economies. 2nd International Symposium on Sustainable Development.

[3] Europe–India SME Business Council [Internet] (2014). Definition of Indian SMEs. http://www.eisbc.org/Definition_of_Indian_SMEs.aspx.

[4] Ministry of Micro Small and Medium Enterprises of India. (a) Growth and performance of MSMEs and (b) 4th Census of MSMEs. Annual Report, 2010–2011; Chapter II: pp. 11–26.

[5] Ministry of Micro, Small and Medium Enterprises, Govt. of India. Growth and performance of MSME sector and (b) Census. Annual Report, 2012–2013; Chapter II: pp. 11–36.

[6] Daǧdeviren M., Yüksel İ (2008). Developing a fuzzy analytic hierarchy process (AHP) model for behavior-based safety management. Inform Sci.

[7] European Agency for Safety and Health at Work (2001). Preventing accidents at work. https://osha.europa.eu/en/publications/magazine/4/.

[8] Beriha G.S., Patnaik B., Mahapatra S.S., Padhee S. (2012). Assessment of safety performance in Indian industries using fuzzy approach. Expert Syst Appl.

[9] Didonet S.R., Díaz G. (2012). Supply chain management practices as a support to innovation in SMEs. J Technol Manag Innov.

[10] World Health Organization (WHO) (1994). Global strategy on occupational health for all: the way to health at work.

[11] Griffin M.A., Neal A. (2000). Perceptions of safety at work: a framework for linking safety climate to safety performance, knowledge, and motivation. J Occup Health Psychol.

[12] Koster R.B.M., Stam D., Balk B.M. (2011). Accidents happen: the influence of safety-specific transformational leadership, safety consciousness, and hazard reducing systems on warehouse accidents. J Oper Manag.

[13] Komaki J., Heinzmann A.T., Lawson L. (1980). Effect of training and feedback: component analysis of behavioral safety program. J Appl Psychol.

[14] Barling J., Loughlin C., Kelloway E.K. (2002). Development and test of a model linking safety-specific transformational leadership and occupational safety. J Appl Psychol.

[15] Komaki J., Barwick K.D., Scott L.R. (1978). A behavioral approach to occupational safety: pinpointing and reinforcing safe performance in a food manufacturing plant. J Appl Psychol.

[16] Cooper M., Philips R., Sutherland V.J., Makin P.J. (1994). Reducing accidents using goal setting and feedback: a field study. J Occup Organ Psychol.

[17] Krause T.R., Seymour K.J., Sloat K.C.M. (1999). Long term evaluation of a behavior-based method for improving safety performance: a meta-analysis of 73 interrupted time-series replications. Safety Sci.

[18] Cox S., Jones B., Rycraft H. (2004). Behavioral approaches to safety management within UK reactor plants. Safety Sci.

[19] Lu C.S., Yang C.S. (2010). Safety leadership and safety behavior in container terminal operations. Safety Sci.

[20] O'Dea A., Flin R. (2001). Site managers and safety leadership in the offshore oil and gas industry. Safety Sci.

[21] Federal Safety Commissioner. Leaders in Safety: A Guide to Developing Senior Management Safety Behaviours in the Building and Construction Industry (Australia): Department of Employment: 2007. Report No. 2 ISBN 978-0-642-32650-8.

[22] Zohar D., Hofmann D.A., Tetrick L.E. (2003). The influence of leadership and climate on occupational health and safety. Health and safety in organizations.

[23] Keeloway E.K., Mullen J., Francis L. (2006). Divergent effects of transformational and passive leadership on employee safety. J Occup Health Psycho.

[24] Wu T.C., Chen C.H., Li C.C. (2007). Correlation among safety leadership, safety climate and safety performance. J Loss Prev Process Ind.

[25] Cooper M.D. (2000). Towards a model of safety culture. Safety Sci.

[26] INSAG (1988). Basic safety principles for nuclear power plants (safety series No 75- INSAG-3).

[27] Glendon A.I., Stanton N.A. (2000). Perspectives on safety culture. Safety Sci.

[28] Baram M., Schoebel M. (2007). Safety culture and behavioral change at the workplace. Safety Sci.

[29] Pousette A., Larsson S., Torner M. (2008). Safety climate cross-validation, strength and prediction of safety behaviour. Safety Sci.

[30] Zohar D. (1980). Safety climate in industrial organizations: theoretical and applied implications. J Appl Psychol.

[31] De Dobbeleer N., Beâland F. (1991). A safety climate measure for construction sites. J Safety Res.

[32] Cox S., Cox T. (1991). The structure of employee attitudes to safety: an European example. Work Stress.

[33] Cooper M.D., Philips R.A. (1994 January). Validation of a safety climate measure. Annual occupational psychology conference.

[34] Cabrera D.D., Isla R., Vilela L.D., Soekkha H.M. (1997). An evaluation of safety climate in ground handling activities. Proceedings of the IASC-97 International Aviation Safety Conference on aviation safety, Netherlands.

[35] Williamson A.M., Feyer A.M., Cairns D., Biancotti D. (1997). The development of a measure of safety climate: the role of safety perceptions and attitudes. Safety Sci.

[36] Brown R.L., Holmes H. (1986). The use of a factor-analytic procedure for assessing the validity of an employee safety climate model. Accident Anal Prev.

[37] Pidgeon N.F. (1991). Safety culture and risk management in organizations. J Cross Cult Psychol.

[38] International Safety Advisory Group (1991). Safety culture.

[39] Glennon D.P. (1982). Measuring organisational safety climate.

[40] Glennon D.P. (1982). Safety climate in organisations.

[41] Ostrom L., Wilhelmsen C., Kaplan B. (1993). Assessing safety culture. Nucl Safety.

[42] Zohar D., Mondelo P., Mattila M., Karwowski W., Hale A. (2006). Safety climate and culture workshop. Proceedings of the 4th International Conference on Occupational Risk Prevention. Seville.

[43] Carrillo R.A., Simon S.I. (1999). Leadership skills that shape and keep world-class safety cultures.

[44] Barua A. (2013). Methods for decision-making in survey questionnaires based on Likert scale. J Asian Sci Res.

[45] Li Q. (2013). A novel Likert scale based on fuzzy sets theory. Exp Syst Appl.

[46] DeJoy D.M. (2005). Behavior change versus culture change: divergent approaches to managing workplace safety. Safety Sci.

[47] Cohen A. (1977). Factors in successful occupational safety programs. J Safety Res.

[48] Paté-Cornell M.E. (1990). Organizational aspects of engineering system safety: the case of offshore platforms. Science.

[49] McAfee R.B., Winn A.R. (1989). The use of incentives/feedback to enhance workplace safety: a critique of the literature. J Safety Res.

[50] Johnston J.J., Cartledge G.T.H., Collins J.W. (1994). The efficacy of training for occupational injury control. Occup Med.

[51] Hofmann D.A., Stetzer A. (1998). The role of safety climate and communication in accident interpretation: implications for learning from negative events. Acad Manage J.

[52] Kaila H.L. (2008). BBS winning over employees in India. [Internet] Occupational Health & Safety. http://ohsonline.com/articles/2008/12/bbs-winning-over-employees-in-india.aspx.

[53] Kaila H.L. (2010). Behavior-based safety programs improve worker safety in India. Ergon Des.

[54] Attock Refinery Team (2008). Behavioural safety at workplace. [Internet] Triple Bottom Line. http://www.tbl.com.pk/behavioural-safety-at-workplace/.

